# General practitioners’ perspectives on data linkage in Australian general practice

**DOI:** 10.23889/ijpds.v9i5.1674

**Published:** 2024-09-18

**Authors:** Heidi Green, Justin Beilby, Felicity Flack, Caroyln Adams, Belinda Fabrianesi, Lucy Carolan, Anthony Brown, Alberto Nettel Aguirre, Annette Braunack-Mayer

**Affiliations:** 1 Australian Centre for Health Engagement, Evidence and Values, School of Health and Society, University of Wollongong; 2 School of Health and Society, University of Wollongong; 3 Population Health Research Network; 4 Macquarie University; 5 Health Consumers NSW; 6 University of Wollongong

## Objective

In the rapidly evolving landscape of digital health, data sharing using primary health care records has gained prominence. Linking primary care data with other administrative health datasets has the potential to provide a whole-of-life perspective on the patient journey and improve health outcomes. This study aimed to gain insight into Australian General Practitioners' (GPs) perspectives on the use of general practice health records for research and data linkage.

## Approach

The study employed a mixed-methods approach. We conducted semi-structured interviews with GPs who had little experience of data sharing, focusing on understanding the challenges and opportunities that increased data sharing poses. We also conducted a three-round modified Delphi study with GPs with experience of and interest in research and data sharing to refine recommendations for data sharing using general practice data.

## Results

In this paper we describe the findings from both arms of the study and compare their recommendations to contribute to a nuanced understanding of general practitioners’ views on data linkage and sharing in general practice.

## Conclusions and Implications

Bridging the knowledge gap between novices and expert practitioners, the findings will inform policies, practices, and initiatives to address the ethical, legal and social implications of data sharing in general practice settings in Australia.

